# A high-protein total diet replacement increases energy expenditure and leads to negative fat balance in healthy, normal-weight adults

**DOI:** 10.1093/ajcn/nqaa283

**Published:** 2020-11-18

**Authors:** Camila L P Oliveira, Normand G Boulé, Arya M Sharma, Sarah A Elliott, Mario Siervo, Sunita Ghosh, Aloys Berg, Carla M Prado

**Affiliations:** Human Nutrition Research Unit, Department of Agricultural, Food, & Nutritional Science, University of Alberta, Edmonton, Alberta, Canada; Alberta Diabetes Institute, University of Alberta, Edmonton, Alberta, Canada; Alberta Diabetes Institute, University of Alberta, Edmonton, Alberta, Canada; Faculty of Kinesiology, Sport, and Recreation, University of Alberta, Edmonton, Alberta, Canada; Division of Endocrinology & Metabolism, Department of Medicine, University of Alberta, Edmonton, Alberta, Canada; Human Nutrition Research Unit, Department of Agricultural, Food, & Nutritional Science, University of Alberta, Edmonton, Alberta, Canada; Alberta Research Centre for Health Evidence, University of Alberta, Edmonton, Alberta, Canada; School of Life Sciences, Division of Physiology, Pharmacology and Neuroscience, University of Nottingham, Nottingham, United Kingdom; Department of Medical Oncology, University of Alberta, Edmonton, Alberta, Canada; Faculty of Medicine, University of Freiburg, Freiburg, Germany; Human Nutrition Research Unit, Department of Agricultural, Food, & Nutritional Science, University of Alberta, Edmonton, Alberta, Canada; Alberta Diabetes Institute, University of Alberta, Edmonton, Alberta, Canada

**Keywords:** protein, total diet replacement, energy metabolism, metabolic biomarkers, adults

## Abstract

**Background:**

High-protein diets and total diet replacements are becoming increasingly popular for weight loss; however, further research is needed to elucidate their impact on the mechanisms involved in weight regulation.

**Objective:**

The aim of this inpatient metabolic balance study was to compare the impact of a high-protein total diet replacement (HP-TDR) versus a control diet (CON) on select components of energy metabolism in healthy adults of both sexes.

**Methods:**

The acute intervention was a randomized, controlled, crossover design with participants allocated to 2 isocaloric arms: *1*) HP-TDR: 35% carbohydrate, 40% protein, and 25% fat achieved through a nutritional supplement; 2) CON: 55% carbohydrate, 15% protein, and 30% fat. Participants received the prescribed diets for 32 h while inside a whole-body calorimetry unit (WBCU). The first dietary intervention randomly offered in the WBCU was designed to maintain energy balance and the second matched what was offered during the first stay. Energy expenditure, macronutrient oxidation rates and balances, and metabolic blood markers were assessed. Body composition was measured at baseline using DXA.

**Results:**

Forty-three healthy, normal-weight adults (19 females and 24 males) were included. Compared with the CON diet, the HP-TDR produced higher total energy expenditure [(EE) 81 ± 82 kcal/d, *P* <0.001], protein and fat oxidation rates (38 ± 34 g/d, *P* <0.001; 8 ± 20 g/d, *P* = 0.013, respectively), and a lower carbohydrate oxidation rate (–38 ± 43 g/d, *P* <0.001). Moreover, a HP-TDR led to decreased energy (–112 ± 85 kcal/d; *P* <0.001), fat (–22 ± 20 g/d; *P* <0.001), and carbohydrate balances (–69 ± 44 g/d; *P* <0.001), and increased protein balance (90 ± 32 g/d; *P* <0.001).

**Conclusions:**

Our primary findings were that a HP-TDR led to higher total EE, increased fat oxidation, and negative fat balance. These results suggest that a HP-TDR may promote fat loss compared with a conventional isocaloric diet. These trials were registered at clinicaltrials.gov as NCT02811276 and NCT03565510.

## Introduction

Total diet replacements (TDRs) are nutritionally complete formula foods designed to replace the whole diet for a specific period of time. In the context of obesity, they may facilitate weight loss. Considering the prevalence of obesity worldwide and its impact on the population's health (1), TDRs are becoming increasingly popular as a weight management strategy; however, research around this topic has not kept pace with its growth in popularity. To our knowledge, only a few studies have evaluated the effects of TDRs in humans to date (2–9). Studies were mostly long-term intervention trials with all participants presenting with obesity (2–9) and sometimes with type 2 diabetes (2, 3, 5, 8, 9). Interventions consisted of calorie-restricted TDRs and the primary outcome was mainly weight loss, which might have influenced all other variables assessed (2–9). None of these studies examined energy metabolism, only some measured metabolic blood markers (2, 5, 7–9), and no studies looked at potential sex differences.

Another potential dietary strategy for body weight management is manipulation of macronutrient intake, particularly high-protein (HP) diets. These diets have gained popularity over the years and their main characteristic is a protein content above recommended values (i.e., for healthy adults aged  >19 y: 0.80 g/kg of body weight/d or 10–35% of total energy intake) (10) with varying levels of carbohydrate and fat intake. High-protein diets are known to increase satiety, energy expenditure (EE), and maintain or increase fat-free mass (FFM), which altogether have been shown to positively affect body weight loss and maintenance (11).

Taken together, the benefits offered by TDR and HP diets seem to be an interesting combination for weight management. Not surprisingly, these synergistic effects have been noticed by industry and several HP-TDR products are widely available to consumers. Although some well-designed inpatient metabolic studies have already assessed the effects of HP diets on energy and substrate metabolism in healthy individuals (12–16), to our knowledge, no inpatient metabolic balance studies have evaluated the exact role of a liquid TDR with an increased protein content on EE, macronutrient oxidation rates and balances, and metabolic blood markers. Additionally, and of extreme importance is the study of this intervention using state-of-the-art methodology in a controlled environment in healthy females and males with a normal body weight to eliminate the confounding effects of obesity and comorbidities on the results. Therefore, the aim of this inpatient metabolic balance study was to compare the impact of a HP-TDR versus a control (CON) diet (North American) on EE, macronutrient oxidation rates and balances, and metabolic blood markers in healthy female and male adults. The primary outcome evaluated was the difference in fat balance between the HP-TDR and CON diets; the secondary outcome was difference in the total EE, with remaining variables as exploratory. It was hypothesized that, compared with the CON diet, participants consuming the HP-TDR would be in negative fat balance, have increased EE, and improved metabolic profile. It was also hypothesized that females and males would respond similarly to the dietary interventions in spite of known sex-related physiological differences.

## Methods

### Study design and ethical procedures

Details of this study protocol have been previously described (17). Briefly, these were 2 complementary randomized, controlled, crossover inpatient studies conducted separately by sex between November 2016 and November 2019 at the Human Nutrition Research Unit (HNRU), University of Alberta (Edmonton, Alberta, Canada). Trial protocols were approved by the University of Alberta Ethics Board (Pro00066006 and Pro00083005) and registered as NCT02811276 (18) and NCT03565510 (19) on clinicaltrials.gov. The studies complied with the standards as set out in the Canadian Tri-Council Policy statement on the use of human participants in research (20). Before study commencement, participants were informed of procedures and potential risks involved in the investigation and provided written informed consent.

### Subjects

Healthy adults, aged 18–35 y, nonsmokers, with BMI between 18.5 and 24.9 kg/m^2^ were recruited via advertisements placed on noticeboards at the University of Alberta. Major exclusion criteria were the presence of any acute or chronic disease, the use of medications and/or nutritional supplements that affect energy metabolism or body composition (e.g., antidepressants, corticosteroids, thyroid disorder medications, creatine and protein supplements), dietary restrictions (e.g., food allergies and/or intolerances and vegetarianism), engagement in exercise practice >1 h/d or 7 h/wk, recent exposure to tests involving radiation, claustrophobia, and specifically for females, pregnancy or lactation and irregular menstrual cycle.

### Experimental protocol

**FIGURE 1:**
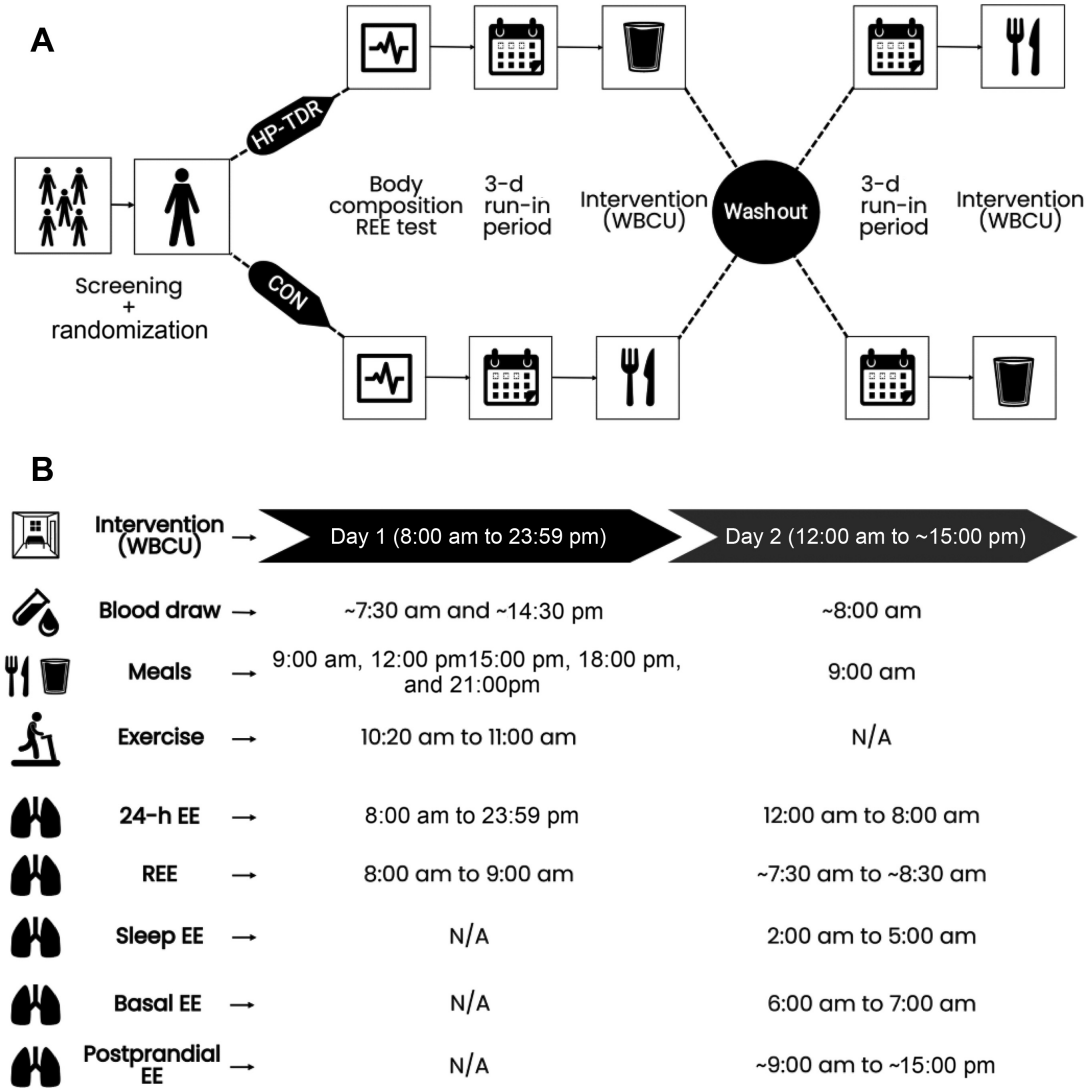
Overview of the experimental protocol (A) and variables assessed during each 32-h test (B). CON, control diet; EE, energy expenditure; HP-TDR, high-protein total diet replacement; N/A, not applicable; REE, resting energy expenditure; WBCU, whole-body calorimetry unit.

Potential participants were instructed to report to the HNRU for a screening visit and, once deemed eligible, were randomly assigned to a HP-TDR or CON diet (1:1) following a simple randomization procedure separated by sex. Following the screening process, eligible participants had their body composition and resting EE (REE) assessed. After these tests, participants underwent 32-h whole-body calorimetry unit (WBCU) assessments for the measurement of energy metabolism components and metabolic blood markers while consuming a eucaloric diet, which was repeated at the second visit (when they crossed-over to the other diet). A eucaloric 3-d run-in diet preceded both intervention phases and was estimated as explained later in this section. Each intervention phase was followed by a washout period of ∼1 mo for females and 2 wk for males. A brief description is presented below and illustrated in **Figure 1**, and fully presented elsewhere (17).

### Anthropometrics and body composition

At baseline, height, weight, waist circumference, and body composition were assessed. Body composition was assessed via DXA using a GE Lunar iDXA (General Electric Company; enCORE software 13.60 Lunar iDXA GE Health Care®). Whole-body and regional levels of fat mass (FM), lean soft tissue (LST), and bone mineral content (BMC) were assessed.

### Study diets

The 3-d individualized run-in diet offered prior to both 32-h WBCU conditions included 3 meals (breakfast, lunch, and dinner) and 2 snacks (afternoon and evening) per day. Participants were instructed to drink water ad libitum, not consume caffeinated food products, or perform strenuous physical activity during this period. The run-in diet provided 55% of total energy intake from carbohydrate, 15% from protein, and 30% from fat.

During both 32-h WBCU stays, 3 meals (breakfast, lunch, and dinner) and 2 snacks (afternoon and evening) were provided on day 1 and 1 meal (breakfast) on day 2 (food items fully described in our protocol article) (17). Bottled water was provided *ad libitum*. The CON diet was comprised of standard food items and the HP-TDR diet consisted of a soy-protein nutritional supplement (Almased®, Almased USA) mixed with olive oil and low-fat milk (1% fat) for the main meals and with olive oil and apple juice for the snacks, per label instructions (21). The nutritional information and ingredient list of the nutritional supplement is described in **Supplementary Table 1**. The first dietary intervention randomly offered in the WBCU was designed to maintain participants in energy balance and the energy content of each meal and snack were similar for the HP-TDR and CON diets (isocaloric). The nutrient content of the dietary interventions is described in **Table 1**.

**FIGURE 3:**
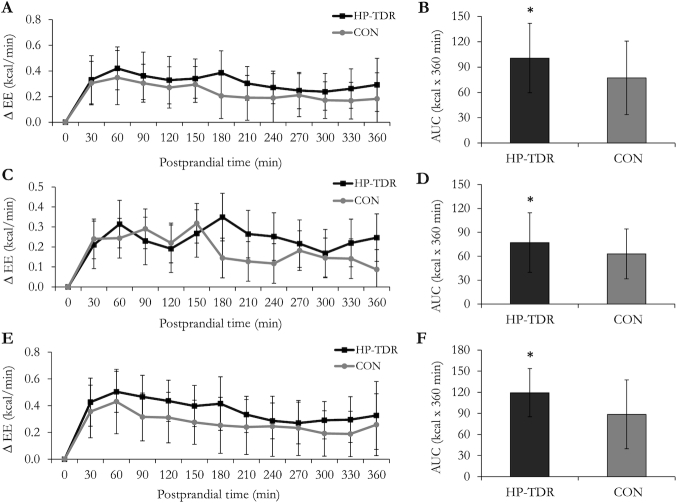
Change in resting energy expenditure (∆ EE) following ingestion of the isocaloric HP-TDR and CON breakfasts on the second day of intervention while participants were inside the whole-body calorimetry unit. Values are mean ± SD. Left panels (A, C, and E) indicate 30-min means; right panels (B, D, and F) indicate the total AUC over 360 minutes. Top panels (A and B) contain data from all participants (*n* = 43); middle panels (C and D) contain data from females (*n* = 19); and bottom panels (E and F) contain data from males (*n* = 24). *Significant difference between the HP-TDR and CON conditions, *P* <0.05 as assessed by a mixed ANOVA. Although there was no statistically significant interaction between the interventions and sex on the total AUC (*P* = 0.115), the main effect of sex showed a significant difference in females and males (*P*  = 0.003), as assessed by a mixed analysis of variance. CON, control; HP-TDR, high-protein total diet replacement.

**TABLE 1:** Nutrient content of the intervention diets

	HP-TDR	CON
Energy, kcal/d	2129 ± 241	2128 ± 241
Protein		
% energy	39.9 ± 0.3	15.3 ± 0.3
g/d	211 ± 24	83 ± 9
Fat		
% energy	24.9 ± 0.3	30.2 ± 0.3
g/d	58 ± 6	72 ± 8
Carbohydrate		
% energy	35.2 ± 0.3	54.4 ± 0.4
g/d	186 ± 21	295 ± 34
Sugars, g/d	179 ± 21	92 ± 12
Fiber, g/d	4 ± 0	30 ± 3
Saturated fat, g/d	12 ± 1	17 ± 3
Monounsaturated fat, g/d	35 ± 3	31 ± 4
Polyunsaturated fat, g/d	5 ± 0	17 ± 2
Cholesterol, mg/d	38 ± 9	107 ± 39

Data are expressed as mean ± SD.

*N* = 43 (*N* = 19 females, *N* = 24 males).

CON, control; HP-TDR, high-protein total diet replacement.

### Energy metabolism

Energy expenditure and macronutrient oxidation rates and balances were assessed by indirect calorimetry measuring the volume of oxygen (VO_2_) and carbon dioxide (VCO_2_), with the use of an open-circuit WBCU. This equipment had a geometric volume of 28.74 m^3^ and was equipped with oxygen (Oxymat, Siemens AG) and carbon dioxide (Advance Optima AO2000 Series, ABB Automation GmbH) analyzers. The information on the volume of gases from the analyzers was then transmitted to a computer (Acer Aspire AM3910-E3122, Acer Inc.) via the National Instruments NI USB-6221 device (National Instruments Corporation) using the PMCSS Software version 1.8 (Pennington Metabolic Chamber Software Suite, Pennington Biomedical Research Center). A 1-h REE indirect calorimetry test was performed at baseline; then, two 32-h tests were conducted while participants consumed the HP-TDR and CON diets. The baseline REE test was used to estimate participant's energy requirements for the 3-d run-in diet and 32-h WBCU tests. To do that, REE was multiplied by a physical activity coefficient, according to the Dietary Reference Intakes (10), and a coefficient of 1.075 representing the metabolizable energy content of the diet (22). The morning following the 3-d run-in periods, participants returned to the HNRU after an 8–12 h overnight fast and spent 32 consecutive hours in the WBCU while receiving the HP-TDR and CON diets in random order, Figure 1A. Both 32-h WBCU tests occurred during the follicular phase of women's menstrual cycle. Throughout each test, blood was drawn 3 times, and urine was collected for the entire time. On the morning of the first day of the test (10:20), a 40-min moderate walking session on a treadmill (BH Fitness T8 SPORT, BH Fitness) was completed, at a personalized fixed pace. Sleep was only allowed during the night.

Total EE and macronutrient oxidation rates were calculated from the measurements of VO_2_, VCO_2_, and urinary nitrogen (N) by using the formula of Brouwer (23). Energy and macronutrient balances were calculated as the difference between intake and oxidation. The respiratory exchange ratio (RER) was calculated as the average ratio of VCO_2_ to VO_2_ per minute during measurements of total EE, REE, basal EE, sleep EE, and postprandial EE. During each WBCU stay, the following EE components were assessed: total EE, REE, basal EE, sleep EE, and postprandial EE, Figure 1B. Diet-induced thermogenesis and arousal EE were not assessed in this study. An internally conducted reliability study for our WBCU (results not published) revealed CVs of 2.2% for total EE, 2.1% for basal EE, and 2.0% for 24-h RER.

### Blood and urine analysis

Blood was sampled by venipuncture at 3 time points during each WBCU stay through an iris port: *1*) the morning on the first day of the test (fasting day 1, ∼07:30); *2*) 2 h after lunch (postprandial, ∼ 14:30); and *3*) the morning on the second day of the test (fasting day 2, ∼08:00). Both morning blood draws were sampled from participants after a 10–12-h overnight fast. Serum samples were analyzed for glucose, insulin, lipid panel (total cholesterol, HDL cholesterol, LDL cholesterol, triglyceride, and non-HDL cholesterol) by DynaLIFE Medical Labs. Plasma samples of free and non-esterified fatty acids (NEFA) were analyzed in-house at the HNRU. The CV in females and males was 1.00% for glucose, 5.00% for insulin, 2.00% for total, HDL, LDL, and non-HDL cholesterol, 3.00% for triglyceride, 7.44% for glycerol, and 6.26% and 9.18% for NEFA in females and males, respectively. HOMA of β-cell function (%B) and insulin resistance (IR) were calculated using the HOMA2 Calculator (©Diabetes Trials Unit, University of Oxford, version 2.2.3). Urine was collected during the entire time participants were in the WBCU for the measurement of total urinary N, which was determined by chemiluminescence using a high temperature Shimadzu TOC-L CPH Model Total Organic Carbon Analyzer with an ASI-L autosampler and TNM-L unit (Shimadzu Corporation, October 2015).

### Statistical analysis

An *a priori* sample size estimation was conducted separately for each sex and is fully described elsewhere (17). Briefly, an effect size of 1.41 was detected with a total of 12 participants per sex based on differences in respiratory quotient from a previously published study (24). In this previous study, individuals receiving a HP-TDR presented a lower value (0.85 ± 0.03) compared with the ones maintaining their usual dietary intake (0.90 ± 0.03) (24). To account for possible dropouts, a total of 14 participants per sex would be required (88% power, α = 0.05) to complete the study. The sample size calculation was done using PASS (Power Analysis and Sample Size software version 19.0.1; NCSS Statistical Software). In addition to the *a priori* sample size estimation, a posthoc analysis was performed and the achieved power (1−β) calculated with the assistance of the G-Power® software (version 3.1.9.7). The power of this study was found to be 99.9% based on a difference in fat balance (i.e., primary outcome) of –22 ± 20 g/d between the HP-TDR and CON groups using a 2-tailed test with an effect size of –1.10, a type I error probability of 0.05, and *n* = 43.

Data were expressed as mean ± SD for continuous variables and frequency and proportions for categorical variables. Mean ± SEM was used to report differences between sexes. Independent t-tests were used to compare the mean differences of continuous variables between sexes at baseline. If the continuous variables were nonnormally distributed, Mann–Whitney U-tests were used to compare the means between the sexes. Chi-square tests were used to correlate 2 categorical variables and Fisher's exact test was used if the cell frequencies were less than a count of 5. Possible differences between the HP-TDR and CON diets were explored using a mixed ANOVA with within-subject factors (i.e., dietary interventions and/or time) and between-subject factors (i.e., sex and/or order of treatment). Posthoc analyses were applied with all ANOVA tests using a Tukey test (equal variances assumed) or Games–Howell (equal variances not assumed). Diagnostics, such as assessing the normality of data, homogeneity of variances using the Box's test of equality of covariance matrices and Levene's test for equality of variances were used to check if the ANOVA assumptions were valid. If the ANOVA assumptions were not met, the corresponding variable was LOG transformed and the ANOVA analysis repeated. A Pearson's product-moment correlation was run to assess the relation between continuous variables and Spearman's Rho was used for non-normally distributed data. Simple regression analysis was used to express total EE, sleep EE, and REE on day 2 as a function of FFM. IBM^®^ SPSS^®^ Statistics version 24 (International Business Machines Corporation) was used to perform all statistical analyses. Differences were regarded as statistically significant if *P* <0.05.

## Results

### Subjects

**FIGURE 2:**
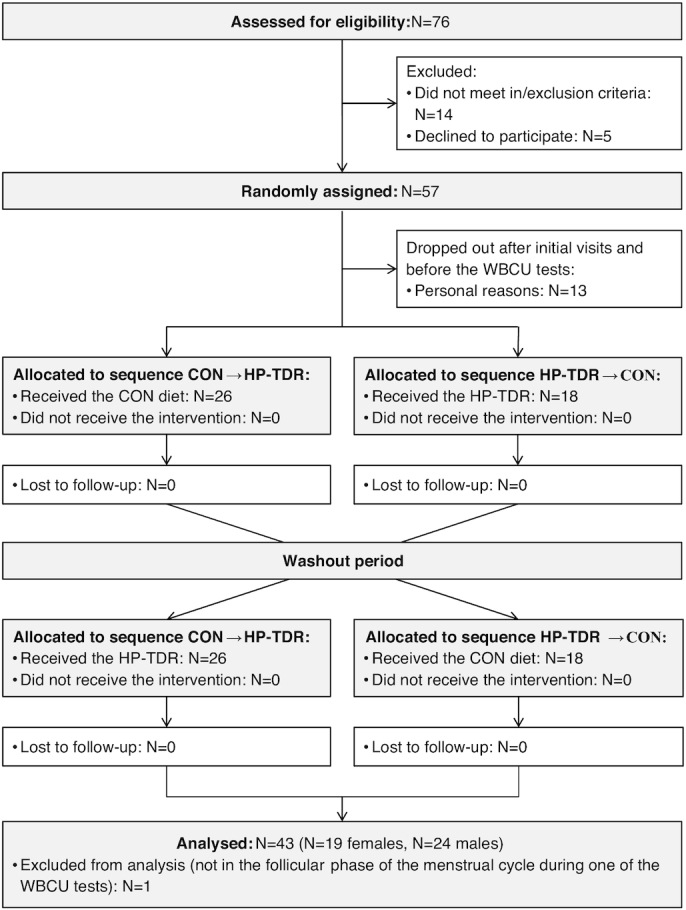
CONSORT flow diagram for crossover trials. CON, control diet; CONSORT, Consolidated Standards of Reporting Trials; HP-TDR, high-protein total diet replacement; WBCU, whole-body calorimetry unit.

Of the 76 potential participants who were screened, 14 (18%) did not meet the eligibility criteria and 5 (6%) declined to participate. Fifty-seven participants were enrolled in the study; 13 (23%) dropped out before the first WBCU test, due to personal reasons. Forty-four participants completed the study (both WBCU tests, *n* = 20 females; *n* = 24 males). One female was excluded from analysis because she was not in the follicular phase of the menstrual cycle during the second WBCU test, **Figure 2**. No adverse events were reported during the study. Baseline characteristics of those who completed the study are summarized in **Table 2**. Compared with males, females were shorter (–8.6 ± 1.8 cm; *P* <0.001), had lower body weight (–5.9 ± 1.8 kg; *P* = 0.003), smaller waist circumference (–5.4 ± 1.4 cm; *P* = 0.001), higher FM (5.8 ± 1.3 kg; *P* <0.001), lower LST (–11.2 ± 1.5 kg; *P* <0.001), lower BMC (–0.4 ± 0.8 kg; *P* <0.001), and lower blood concentrations of albumin (–3 ± 1 g/L; *P* <0.001), creatinine (–17 ± 3 mmol/L; *P* <0.001), and sodium (–2 ± 1 mmol/L; *P* <0.001).

**TABLE 2:** Baseline characteristics of the study participants

Characteristics	All (*n* = 43)	Females (*n* = 19)	Males (*n* = 24)	Sex difference^1^
Age, y	24 ± 4	25 ± 3	23 ± 4	0.090
Height, cm	171.1 ± 7.3	166.3 ± 5.7	174.9 ± 6.1	<0.001
Weight, kg	64.4 ± 6.9	61.1 ± 4.8	67.0 ± 7.3	0.003
Waist circumference, cm	74.4 ± 5.6	71.4 ± 2.8	76.9 ± 6.1	0.001
BMI, kg/m^2^	22.0 ± 1.4	22.2 ± 1.2	21.9 ± 1.6	0.522
FM, kg	15.3 ± 5.1	18.6 ± 3.3	12.7 ± 4.9	<0.001
LST, kg	46.4 ± 7.6	40.1 ± 4.4	51.4 ± 5.6	<0.001
BMC, kg	2.7 ± 0.3	2.4 ± 0.2	2.9 ± 0.3	<0.001
Race				0.202
White	19 (25)	7 (26)	12 (50)	
Asian	14 (27)	5 (28)	9 (26)	
Hispanic	3 (7)	3 (16)	0 (0)	
Black	1 (2)	1 (5)	0 (0)	
Other	6 (14)	3 (16)	3 (13)	
Physical activity level^2^				0.270
Insufficiently active	2 (5)	1 (6)	1 (4)	
Moderately active	7 (16)	5 (28)	2 (8)	
Active	34 (79)	13 (68)	21 (88)	
Medication/nutritional supplement in use				0.412
None	34 (79)	13 (68)	21 (88)	
Multivitamin/mineral	5 (12)	3 (16)	2 (8)	
Antidepressant	3 (7)	2 (11)	1 (4)	
Antihistamine	1 (2)	1 (5)	0 (0)	
Birth control method in use			N/A	N/A
None	9 (21)	9 (47)		
Birth control pills	9 (21)	9 (47)		
Non-hormonal intrauterine device	1 (2)	1 (6)		
Blood markers				
ALT, U/L	22 ± 10	18 ± 5	24 ± 12	0.058
AST, U/L	25 ± 21	21 ± 5	29 ± 27	0.233
Serum albumin, g/L	45 ± 2	43 ± 2	47 ± 2	<0.001
Creatinine, mmol/L	80 ± 13	70 ± 10	88 ± 9	<0.001
Estimated GFR, mL/min/1.73m^2^	106 ± 14	105 ± 15	107 ± 13	0.572
Sodium, mmol/L	141 ± 2	139 ± 2	142 ± 1	<0.001
Potassium, mmol/L	4.3 ± 0.3	4.3 ± 0.3	4.4 ± 0.2	0.177
Chloride, mmol/L	104 ± 2	104 ± 2	104 ± 2	0.742
TSH, mU/L	1.74 ± 0.74	1.90 ± 0.75	1.61 ± 0.72	0.211

Data are expressed as mean ± SD or *n* (%).

1 *P* values refer to differences between females and males. For continuous variables, *P* values were detected with the use of an independent-samples t-test or Mann–Whitney U test, accordingly. For nominal variables, *P* values were detected with the use of the Fisher's exact test.

2 Physical activity levels were classified according to the Godin-Shephard Leisure-­Time Physical Activity Questionnaire.

ALT, alanine aminotransferase; AST, aspartate aminotransferase; BMC, bone mineral content; FM, fat mass; GFR, glomerular filtration rate; LST, lean soft tissue; N/A, not applicable; TSH, thyroid stimulating hormone.

### Energy metabolism

Differences of selected energy metabolism components between the HP-TDR and CON diets are shown in **Table 3**. During the HP-TDR intervention, total and sleep EE were increased by 81 ± 82 kcal/d (*P* <0.001) and 17 ± 26 kcal/8-h night (*P* <0.001), respectively. Resting EE on day 1 (*P* = 0.784), on day 2 (*P* = 0.582), and basal EE (*P* = 0.411) did not differ between diets. While consuming the HP-TDR, 24-h RER was lower (–0.02 ± 0.01; *P* <0.001) compared with the CON diet. The RER during measurements of REE on day 2, basal EE, and sleep EE, were also lower during the HP-TDR diet, *P* <0.001. Carbohydrate oxidation rate was lower during the HP-TDR diet (–38 ± 43 g/d, *P* <0.001), and protein and fat oxidation rates were higher (38 ± 34 g/d, *P* <0.001; 8 ± 20 g/d, *P* = 0.013, respectively). Compared with the CON diet, while consuming a HP-TDR, participants experienced lower carbohydrate (–69 ± 44 g/d; *P* <0.001), fat (–22 ± 20 g/d; *P* <0.001), and energy (–112 ± 85 kcal/d; *P* <0.001) imbalances, and greater protein imbalance (90 ± 32 g/d; *P* <0.001). Moreover, the HP-TDR led to an increased EE above resting (assessed on day 2) following the ingestion of isocaloric breakfasts (day 2 WBCU stay), **Figure 3**.

**TABLE 3:** Energy expenditure, respiratory exchange ratio, and macronutrient oxidation rates and balances during the HP-TDR and CON diets

	HP-TDR			CON					
	All (*n* = 43)	Female (*n* = 19)	Male (*n* = 24)	All (*n* = 43)	Female (*n* = 19)	Male (*n* = 24)	Diet effect^1^	Sex effect^1^	Diet × sex^1^
Total EE, kcal/d	2143 ± 268	1967 ± 195	2283 ± 234	2061 ± 243	1899 ± 143	2189 ± 231	<0.001	<0.001	0.300
24-h RER	0.85 ± 0.02	0.84 ± 0.01	0.86 ± 0.01	0.87 ± 0.01	0.87 ± 0.01	0.87 ± 0.02	<0.001	0.038	0.333
Resting									
REE Day 1, kcal/d	1620 ± 259	1432 ± 138	1768 ± 236	1621 ± 206	1491 ± 115	1724 ± 204	0.784	<0.001	0.067
RER - Day 1	0.81 ± 0.02	0.81 ± 0.03	0.81 ± 0.02	0.82 ± 0.04	0.81 ± 0.03	0.83 ± 0.03	0.213	0.063	0.066
REE Day 2, kcal/d	1612 ± 215	1462 ± 168	1731 ± 169	1605 ± 247	1408 ± 147	1761 ± 193	0.582	<0.001	0.064
RER - Day 2	0.82 ± 0.02	0.81 ± 0.02	0.82 ± 0.02	0.86 ± 0.03	0.85 ± 0.02	0.86 ± 0.03	<0.001	0.242	0.394
Basal									
Basal EE, kcal/d	1584 ± 242	1409 ± 149	1723 ± 211	1600 ± 223	1450 ± 165	1719 ± 191	0.411	<0.001	0.315
RER	0.83 ± 0.02	0.83 ± 0.02	0.84 ± 0.02	0.87 ± 0.02	0.87 ± 0.02	0.87 ± 0.02	<0.001	0.518	0.689
Sleep									
Sleep EE,^3^ kcal/8-h night	498 ± 49	469 ± 47	522 ± 37	481 ± 53	450 ± 44	505 ± 48	<0.001	<0.001	0.864
RER	0.82 ± 0.02	0.82 ± 0.02	0.82 ± 0.01	0.85 ± 0.02	0.85 ± 0.02	0.85 ± 0.02	<0.001	0.664	0.661
Carbohydrate ox, g/d	235 ± 43	219 ± 31	247 ± 47	273 ± 40	256 ± 27	286 ± 45	<0.001	0.007	0.867
Protein ox,^2^ g/d	91 ± 40	67 ± 24	110 ± 40	53 ± 20	36 ± 8	67 ± 16	<0.001	<0.001	0.212
Fat ox, g/d	79 ± 17	78 ± 17	79 ± 16	71 ± 16	69 ± 9	72 ± 20	0.013	0.614	0.729
Carbohydrate balance, g/d	–48 ± 33	–43 ± 25	–51 ± 38	22 ± 26	21 ± 18	23 ± 32	<0.001	0.631	0.463
Protein balance, g/d	119 ± 34	132 ± 23	110 ± 39	29 ± 19	42 ± 6	19 ± 19	<0.001	0.001	0.959
Fat balance, g/d	–20 ± 17	–23 ± 17	–18 ± 16	1 ± 15	–0.6 ± 10	3 ± 18	<0.001	0.246	0.867
Energy balance, kcal/d	–18 ± 113	32 ± 101	–58 ± 109	92 ± 129	164 ± 100	35 ± 121	<0.001	0.001	0.143

Data are presented as mean ± SD.

1 *P* values were detected with the use of a mixed ANOVA.

2 Data were not normally distributed and log-transformed for statistical analysis.

3 Sleep EE reflects an 8-h sleep period.

CON, control; EE, energy expenditure; HP-TDR, high-protein total diet replacement; ox, oxidation; REE, resting energy expenditure; RER, respiratory exchange ratio.

Although no diet × sex interaction was observed in any of the variables assessed (*P* >0.05), a main effect of sex on several energy metabolism variables was detected. Compared with males, females presented lower total EE (–303 ± 62 kcal/d, *P* <0.001), 24-h RER (–0.008 ± 0.004, *P* = 0.038), REE on day 1 (–284 ± 50 kcal/d, *P* <0.001) and on day 2 (–311 ± 48 kcal/d, *P* <0.001), basal EE (–291 ± 51, *P* <0.001), sleep EE (–53 ± 12 kcal/8-h night, *P* <0.001), postprandial EE (–0.2 ± 0.04 kcal/min, *P* 0.001), RER during postprandial EE assessment (–0.01 ± 0.004, *P* = 0.009), carbohydrate oxidation rate (–29 ± 10 g/d, *P* = 0.007), protein oxidation rate (–36 ± 6 g/d, *P* <0.001), and greater protein (22 ± 6 g/d, *P* = 0.001) and energy (109 ± 31 kcal/d, *P* = 0.001) imbalances. More specifically, during each dietary intervention, energy balance was different between sexes (HP-TDR: females 32 ± 23 kcal/d, males –58 ± 22 kcal/d, *P* = 0.008; CON: females 164 ± 23 kcal/d, males 35 ± 25 kcal/d, *P* = 0.001).

**FIGURE 4:**
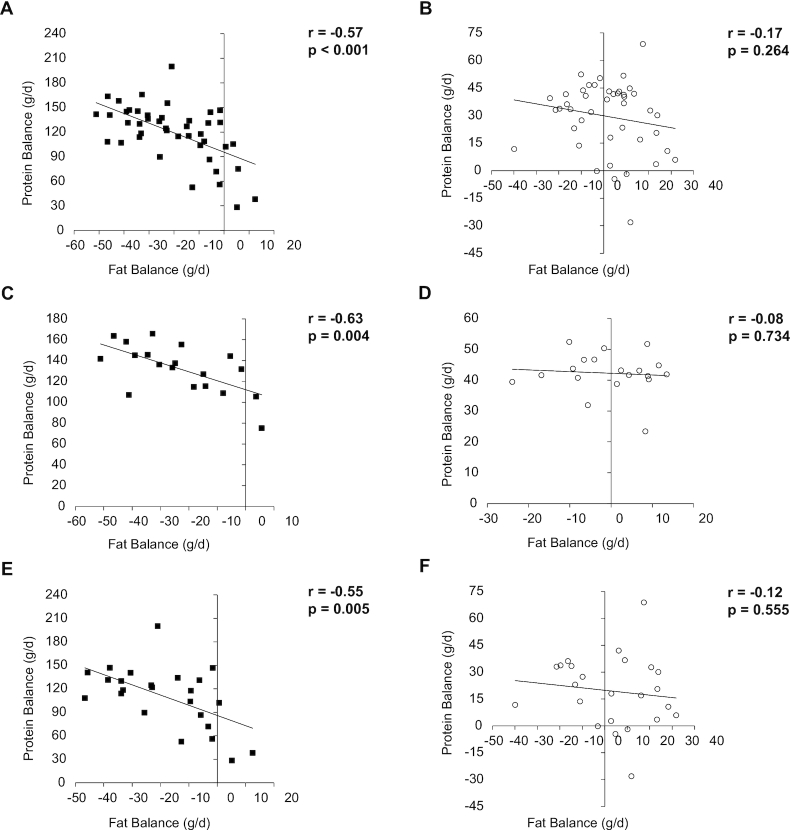
Correlation between protein and fat balances in all participants (*n* = 43, panels A and B), females (*n* = 19, panels C and D), and males (*n*  = 24, panels E and F). Black squares (▓) represent the HP-TDR condition and empty circles (○) represent the CON condition. CON, control, HP-TDR, high-protein total diet replacement.

Protein and fat balances were inversely correlated only in the HP-TDR diet (all: r = –0.57, *P* <0.001; females: r = –0.63, *P* = 0.004; males: r = –0.55, *P* = 0.005), **Figure 4**. Total EE and LST were positively correlated in both diets (HP-TDR: r = 0.79, *P* <0.001; CON: r = 0.79, *P* <0.001). In females and males, total EE, sleep EE, and REE on day 2 were a function of FFM during the HP-TDR and CON conditions, except for sleep EE in females in the HP-TDR (**Supplementary Figures 1** and **2**). The order in which participants received the dietary interventions did not affect any of the energy metabolism variables analyzed (all *P* >0.05).

### Metabolic blood markers

Metabolic blood markers assessed in a fasting state on days 1 and 2, and after lunch during the HP-TDR and CON diets are shown in **Table 4**. Glycerol (–4.2 ± 12.4 µM, *P* = 0.031) and triglyceride (–0.07 ± 0.23 mmol/L, *P* = 0.044) decreased more from fasting day 1 to fasting day 2 in the HP-TDR compared with the CON diet, and total, LDL, and non-HDL cholesterol blood concentrations increased more (0.10 ± 0.26 mmol/L, *P* = 0.010; 0.12 ± 0.18 mmol/L, *P* <0.001; 0.09 ± 0.20 mmol/L, *P* = 0.005, respectively). On the other hand, this change was not different between the dietary interventions for glucose, insulin, HOMA %B, HOMA IR, NEFA, and HDL cholesterol, *P* >0.05.

**TABLE 4:** Metabolic blood markers during the HP-TDR and CON diets

	HP-TDR			CON			∆^1^		Postprandial^2^	
	Fasting day 1	Postprandial	Fasting day 2	Fasting day 1	Postprandial	Fasting day 2	Diet effect	Diet × sex	Diet effect	Diet × sex
Glucose,^3^ mmol/L	4.8 ± 0.3	4.7 ± 0.4	4.7 ± 0.2	4.8 ± 0.3	4.9 ± 0.5	4.9 ± 0.3	0.126	0.502	0.044	0.765
Insulin,^3^ pmol/L	43.1 ± 15.4	62.8 ± 35.1	35.6 ± 13.8	44.8 ± 18.4	81.1 ± 50.2	37.1 ± 15.3	0.804	0.121	0.007	0.359
HOMA %B^3^	89.0 ± 20.5	—	78.7 ± 17.4	88.4 ± 21.7	—	76.2 ± 22.9	0.578	0.362	—	—
HOMA IR^3^	0.8 ± 0.3	—	0.6 ± 0.3	0.8 ± 0.3	—	0.7 ± 0.3	0.716	0.087	—	—
Glycerol,^4^ μM	27.5 ± 19.6	32.3 ± 23.0	19.3 ± 11.4	23.7 ± 14.4	47.6 ± 29.5	19.6 ± 12.0	0.031	0.684	<0.001	0.682
NEFA,^4^ μM	201.2 ± 191.6	115.2 ± 123.4	154.8 ± 139.5	182.1 ± 150.6	104.1 ± 88.4	145.7 ± 132.0	0.880	0.190	0.513	0.160
Lipid panel^3^										
Total cholesterol, mmol/L	4.34 ± 0.73	4.33 ± 0.73	4.43 ± 0.78	4.30 ± 0.69	4.19 ± 0.72	4.28 ± 0.76	0.010	0.286	0.041	0.174
LDL cholesterol, mmol/L	2.41 ± 0.52	2.28 ± 0.49	2.54 ± 0.52	2.38 ± 0.49	2.13 ± 0.5	2.39 ± 0.5	<0.001	0.647	0.023	0.796
HDL cholesterol, mmol/L	1.45 ± 0.43	1.45 ± 0.46	1.44 ± 0.48	1.43 ± 0.43	1.40 ± 0.44	1.40 ± 0.43	0.214	0.042	0.047	0.436
Non-HDL cholesterol, mmol/L	2.89 ± 0.58	2.88 ± 0.52	2.99 ± 0.58	2.88 ± 0.5	2.79 ± 0.52	2.88 ± 0.56	0.005	0.645	0.082	0.258
Triglyceride, mmol/L	1.06 ± 0.42	1.31 ± 0.63	0.98 ± 0.34	1.08 ± 0.41	1.45 ± 0.57	1.08 ± 0.42	0.044	0.958	0.128	0.143

Data are presented as mean ± SD.

1 *P* values represent the main effect of diet on the change from fasting day 1 to fasting day 2 and were detected with the use of a mixed ANOVA.

2 *P* values represent the main effect of diet on postprandial values and were detected with the use of a mixed ANOVA.

3 *N* = 41 (*N* = 17 females, *N* = 24 males).

4 *N* = 42 (*N* = 18 females, N = 24 males).

CON, control; HOMA %B, homeostatic model assessment of β-cell function; HP-TDR, high-protein total diet replacement.

There was a statistically significant interaction between diet and sex on the change in HDL cholesterol concentration (*P* = 0.042). In the HP-TDR diet, the HDL cholesterol concentration was greater in females compared with males (0.08 ± 0.03 mmol/L, *P* = 0.007). Moreover, the change in HDL cholesterol from fasting day 1 to fasting day 2 was significantly different between interventions in females (HP-TDR: 0.03 ± 0.03 mmol/L; CON: –0.01 ± 0.02 mmol/L; *P* = 0.043), but not in males (HP-TDR: –0.04 ± 0.02 mmol/L; CON: –0.05 ± 0.12 mmol/L; *P* = 0.525). There was no difference between sexes in the CON diet (*P* = 0.430).

Postprandially, glucose (–0.2 ± 0.5 mmol/L, *P* = 0.044), insulin (–19.1 ± 44.6 pmol/L, *P* = 0.007), and glycerol (–16.8 ± 25.9 μM, *P* <0.001) blood concentrations were lower in the HP-TDR diet compared with the CON, and total, LDL, and HDL cholesterol concentrations were higher (0.12 ± 0.42 mmol/L, *P* = 0.041; 0.12 ± 0.34 mmol/L, *P* = 0.023; 0.06 ± 0.20 mmol/L, *P* = 0.047, respectively). There was no diet × sex interaction in any of the variables analyzed postprandially (all *P* >0.05).

The order in which participants received the dietary interventions did not affect any of the metabolic blood markers analyzed, *P* >0.05. A diet × sex × time interaction was also explored, and no statistically significant 3-way interaction was observed in any of the variables analyzed (**Supplementary Table 2**).

## Discussion

The present inpatient metabolic balance study compared the effects of an isocaloric HP-TDR versus a CON diet on EE, macronutrient oxidation rates and balances, and metabolic blood markers in female and male healthy adults. The primary findings of this study were that compared with a standard North American dietary pattern, a HP-TDR led to higher total EE, increased fat oxidation, and negative fat balance (likely implying body fat loss) (25). The only diet × sex interaction observed was on HDL cholesterol concentration in the HP-TDR diet. These results highlight the impact HP-TDR consumption has on energy metabolism and metabolic blood markers of healthy adults and provides further insight into the potential role of this dietary strategy for weight management.

Regarding the components of participant's EE, this study showed that consumption of the HP-TDR led to higher daily, sleep, and postprandial EE. Collectively, these results add to the discussion that a calorie is *not* just a calorie (26) and that isocaloric diets with a different proportion of macronutrients might offer a metabolic advantage (27–29), specifically an increase in EE and fat oxidation. There seems to be a consensus that the protein content of the diet can directly affect EE and substrate use (11, 30); however, the same is not true when it comes to the carbohydrate and fat contents (31, 32, 33). It is possible that energetic costs involved in the thermic effect of protein and the possible increase in protein turnover contributed to the observed increase in EE in this group (11, 30), which is concordant with the literature (13). On the other hand, 24 h after the start of the interventions, participant's REE and basal EE did not differ between diets, contrasting previous findings (13). Interestingly, it seems that eucaloric HP diets are not able to change REE as it does with other components of EE, which can only be captured with the sophisticated measurement of energy metabolism (i.e., using a WBCU). Previous studies showing an increase or decline in REE with HP diets were long-term interventions in which participants were in negative or positive energy balance. A meta-analysis of randomized controlled trials revealed that HP diets reduced the decline in REE during weight loss, which has been potentially attributed to a retention of lean mass, although this has not been determined (34). In addition to that, overfeeding a HP diet for 8 wk has been shown to increase REE (227 kcal/d) and this result was associated with an accretion of 3.18 kg of lean mass (14). Due to our experimental design, lean mass and therefore REE were not expected to change, which is in line with current literature.

Over the years, experiments have shown that total body carbohydrate and protein content are tightly regulated by adjusting oxidation rates to intake levels, meaning that manipulating the intake of these macronutrients affects their oxidation rates to the same direction and extent (25, 35, 36). In this study, a HP-TDR led to a decrease in carbohydrate oxidation rate and an increase in protein oxidation rate, which is in line with this rationale since the HP-TDR intervention has a low-carbohydrate, HP content. Conversely, this autoregulatory process is nonexistent for fat oxidation, which seems to be mostly driven by the presence or absence of other macronutrients, markedly carbohydrate (25). The dynamic interactions between carbohydrate and fat oxidation started to be described almost 60 y ago (37) and have been continuously explored as more research is made available (38). As comprehensively discussed by Hue and Taegtmeyer (38) and illustrated by Prentice (25), the low-carbohydrate characteristic of the HP-TDR seems to be responsible for the increased fat oxidation observed with this dietary intervention. This result is further demonstrated by the lower 24-h RER observed in the HP-TDR diet. As a consequence of intake and oxidation rates in this study, participants consuming the HP-TDR experienced a decrease in energy and fat balances, likely implying body fat loss (25). In a classical inpatient experiment, Abbott et al. (36) demonstrated that in conditions of energy imbalance, fat stores are mobilized to balance the body's energy budget, which is in agreement with results presented herein.

In this study, total, LDL, and non-HDL cholesterol blood concentrations increased more from fasting day 1 to fasting day 2 in the HP-TDR compared with the CON diet. Although change in these markers was statistically significant, the absolute values remained within the reference ranges for this population group. Jones et al. (39) demonstrated that the ingestion of dietary cholesterol causes feedback inhibition of cholesterol biosynthesis in humans. Considering that the content of dietary cholesterol of the HP-TDR intervention was almost 3 times lower than the content of the CON diet, it might be possible that the participants' biosynthesis was upregulated in the HP-TDR, causing an increase in blood lipid concentrations. On the contrary, blood triglyceride concentration was lower in the HP-TDR diet compared with the CON diet. This fact can be mainly attributed to the low carbohydrate content of this dietary intervention (40), also supported by previous studies (41). Additionally, blood glycerol decreased more from fasting day 1 to fasting day 2 in the HP-TDR compared with the CON diet. Circulating glycerol has been shown to result mainly from hydrolysis of triglyceride stored in adipose tissue, and constitutes a major substrate for glucose homeostasis (42). The increased fat oxidation and negative fat balance observed in the HP-TDR group are both indicative of increased hydrolysis of triglycerides in adipose tissue, which might have greatly contributed to the use of this substrate as an energy source, reducing its circulating concentrations. A significant interaction between diet and sex on the change in HDL cholesterol concentration was found in the HP-TDR diet, in which females presented greater values compared with males. An analysis of 1.3 million patients revealed that the HDL cholesterol concentration is higher in females than males (43). This effect seems to be related to females' increased endogenous (44) and exogenous estrogens (e.g., estrogen-containing contraceptives) (45). Considering that the HP-TDR contained soy isoflavones (46), which are natural estrogen-like compounds, it might be possible that it could have elevated the females' estrogen concentrations, which accentuated the difference in HDL cholesterol concentration between sexes in the HP-TDR diet.

To date, studies investigating the effects of TDRs have been conducted in individuals with obesity and/or comorbidities in a state of negative energy balance with the main objective of weight loss (2, 4–9). The presence of several confounding variables in these studies, such as weight loss and comorbidities, hinders our understanding of the real physiological impact of TDRs. To our knowledge, this is the first study to compare a HP-TDR with a North American diet in healthy young adults of both sexes. In addition to being the first on the topic, this study has several strengths, including its crossover and rigorously controlled feeding design, allowing the detection of small diet effects on energy metabolism variables and metabolic blood markers. Moreover, the use of state-of-the-art technology, such as the WBCU, provided highly accurate and precise results, reflecting the real effects of the dietary interventions. In addition to the design and technology used, the study of both females and males allowed us to explore how different sexes respond to these dietary interventions.

In this study, participants received isocaloric diets that were designed to mimic the North American dietary pattern and a nutritional product commercially available in many countries. For this reason, 1 or more macronutrients could not be kept constant in 1 dietary intervention while others were manipulated in the other intervention. When comparing the macronutrient distribution of the HP-TDR with the Acceptable Macronutrient Distribution Range (10), this dietary strategy can be characterized as HP and low-carbohydrate. Therefore, it is not possible to attribute any of the results observed in this study to a single macronutrient. In addition, this study has other limitations, including the specificity of the population being studied (i.e., healthy, young adults with a normal body weight) and the short-term intervention. These limitations restrict our ability to translate these results to other population groups and longer intervention periods. Therefore, future studies are needed to better understand the long-term effects of this dietary intervention on the physiology of healthy and diseased population groups.

## Supplementary Material

nqaa283_Supplemental_Tables_Figures

## Notes

This was an investigator-initiated trial supported by Almased Wellness GmbH (Bienenbüttel, Germany). Per contractual agreement, the funder has had no role in the design of the study, in the collection, analysis, and interpretation of data, in the writing of the report, and in the decision to submit the article for publication. CMP is supported by a Canadian Institutes of Health Research New Investigator Salary Award, and a Campus Alberta Innovates Program.

Supplemental Tables 1 and 2 and Supplemental Figures 1 and 2 are available from the “Supplementary data” link in the online posting of the article and from the same link in the online table of contents at https://academic.oup.com/ajcn/.

The authors will make the data (in deidentified form, if human data) used in the manuscript, code book, and analytic code available to editors upon request either before or after publication.

Abbreviations used: %B, β-cell function; BMC, bone mineral content; CON, control; EE, energy expenditure; FM, fat mass; HP, high-protein; HP-TDR, high-protein total diet replacement; HNRU, Human Nutrition Research Unit; IR, insulin resistance; LST, lean soft tissue; N, nitrogen; NEFA, non-esterified fatty acids; REE, resting energy expenditure; RER, respiratory exchange ratio; TDR, total diet replacement; VCO_2_, volume of carbon dioxide; VO_2_, volume of oxygen; WBCU, whole-body calorimetry unit.
