# Corrigendum to: A high-protein total diet replacement increases energy expenditure and leads to negative fat balance in healthy, normal-weight adults. Am J Clin Nutr 2021;113:476–487

**DOI:** 10.1093/ajcn/nqaa391

**Published:** 2021-02-02

Corrigendum to: A high-protein total diet replacement increases energy expenditure and leads to negative fat balance in healthy, normal-weight adults. Am J Clin Nutr 2021;113:476–487.

In the above article, Table 1 has been updated as follows online:

Previous version

**TABLE 1:** Nutrient content of the intervention diets

	HP-TDR	CON
Energy, kcal/d	2129 ± 241	2128 ± 241
Protein		
% energy	39.9 ± 0.3	15.3 ± 0.3
g/d	211 ± 2	83 ± 9
Fat		
% energy	24.9 ± 0.3	30.2 ± 0.3
g/d	58 ± 6	72 ± 8
Carbohydrate		
% energy	35.2 ± 0.3	54.4 ± 0.4
g/d	186 ± 21	295 ± 34
Sugars, g/d	179 ± 21	4.6 ± 0.5
Fiber, g/d	4 ± 0	92 ± 12
Saturated fat, g/d	12 ± 1	29 ± 3
Monounsaturated fat, g/d	35 ± 3	16 ± 2
Polyunsaturated fat, g/d	5 ± 0	31 ± 4
Cholesterol, mg/d	38 ± 9	17 ± 1

Data are expressed as mean ± SD.

*N* = 43 (*N* = 19 females, *N* = 24 males).

CON, control; HP-TDR, high-protein total diet replacement.

Corrected version 

**TABLE 1:** Nutrient content of the intervention diets

	HP-TDR	CON
Energy, kcal/d	2129 ± 241	2128 ± 241
Protein		
% energy	39.9 ± 0.3	15.3 ± 0.3
g/d	211 ± 24	83 ± 9
Fat		
% energy	24.9 ± 0.3	30.2 ± 0.3
g/d	58 ± 6	72 ± 8
Carbohydrate		
% energy	35.2 ± 0.3	54.4 ± 0.4
g/d	186 ± 21	295 ± 34
Sugars, g/d	179 ± 21	92 ± 12
Fiber, g/d	4 ± 0	30 ± 3
Saturated fat, g/d	12 ± 1	17 ± 3
Monounsaturated fat, g/d	35 ± 3	31 ± 4
Polyunsaturated fat, g/d	5 ± 0	17 ± 2
Cholesterol, mg/d	38 ± 9	107 ± 39

Data are expressed as mean ± SD.

*N* = 43 (*N* = 19 females, *N* = 24 males).

CON, control; HP-TDR, high-protein total diet replacement.

