# Supplementary material for: A high-protein total diet replacement increases energy expenditure and leads to negative fat balance in healthy, normal-weight adults
Source: Am J Clin Nutr. 2020 Nov 18;113(2):476–87. doi: 10.1093/ajcn/nqaa283 (PMC7851826; doi:10.1093/ajcn/nqaa283)
Supplement: nqaa283_Supplemental_Tables_Figures [file nqaa283_supplemental_tables_figures.zip › On-line Supplementary Material - Figure 1.pdf]

A high-protein total diet replacement increases energy expenditure and leads to negative fat balance in healthy, normal-weight adults. Camila L. P. Oliveira. Online Supplementary Material.

Supplementary Figure 1. Correlation between fat-free mass (FFM) and total energy expenditure (total EE, panels A and B), sleep energy expenditure (sleep EE, panels C and D), and resting energy expenditure on day 2 (REE, panels E and F) in females (n=19). Black squares (■) represent the high-protein total diet replacement (HP-TDR) condition and empty circles (○) represent the control (CON) condition.

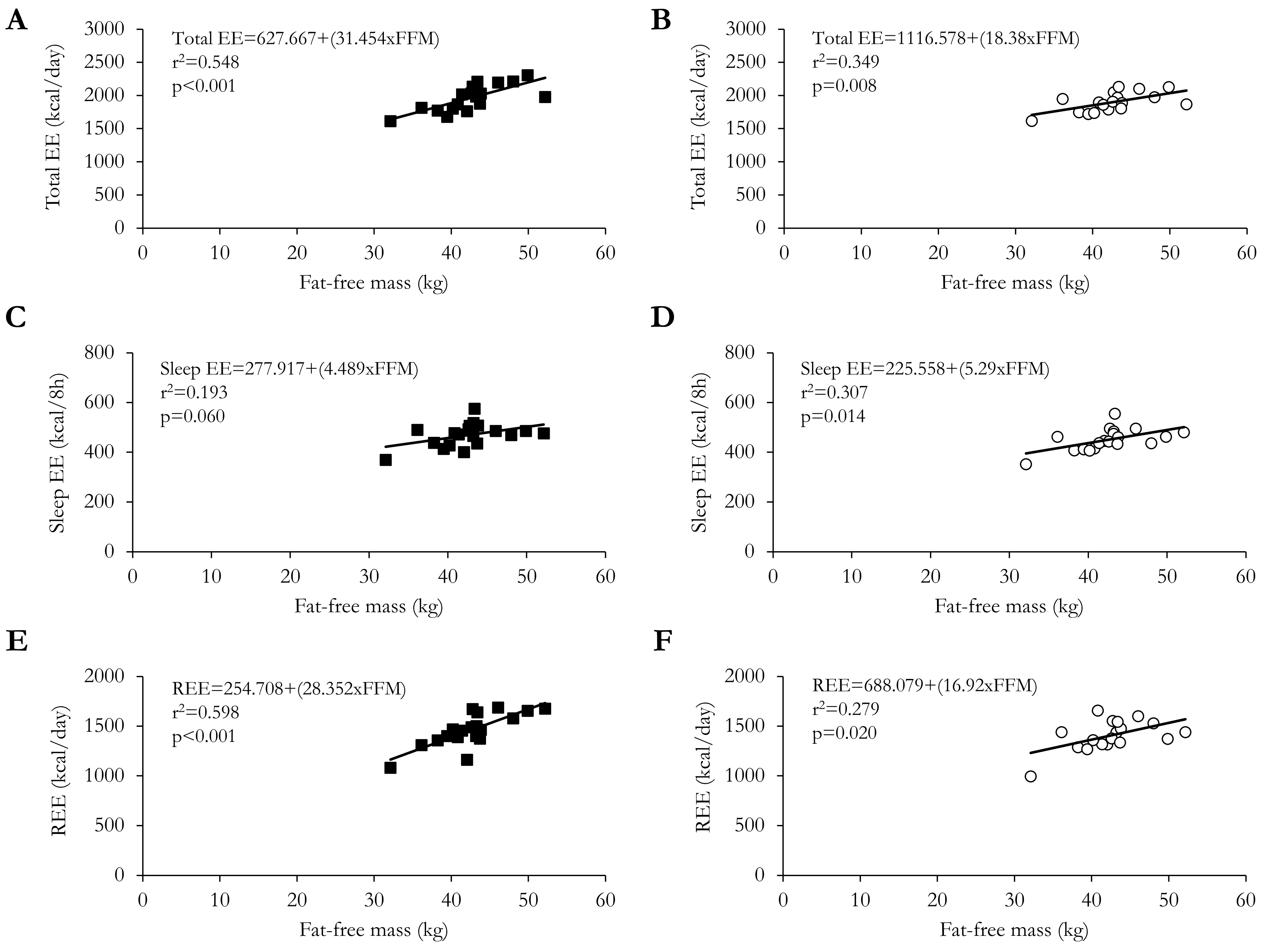
A
Total EE=627.667+(31.454xFFM)
r2=0.548
p<0.001
Total EE (kcal/day)
Fat-free mass (kg)
B
Total EE=1116.578+(18.38xFFM)
r2=0.349
p=0.008
Total EE (kcal/day)
Fat-free mass (kg)
C
Sleep EE=277.917+(4.489xFFM)
r2=0.193
p=0.060
Sleep EE (kcal/8h)
Fat-free mass (kg)
D
Sleep EE=225.558+(5.29xFFM)
r2=0.307
p=0.014
Sleep EE (kcal/8h)
Fat-free mass (kg)
E
REE=254.708+(28.352xFFM)
r2=0.598
p<0.001
REE (kcal/day)
Fat-free mass (kg)
F
REE=688.079+(16.92xFFM)
r2=0.279
p=0.020
REE (kcal/day)
Fat-free mass (kg)
