# Supplementary material for: A high-protein total diet replacement increases energy expenditure and leads to negative fat balance in healthy, normal-weight adults
Source: Am J Clin Nutr. 2020 Nov 18;113(2):476–87. doi: 10.1093/ajcn/nqaa283 (PMC7851826; doi:10.1093/ajcn/nqaa283)
Supplement: nqaa283_Supplemental_Tables_Figures [file nqaa283_supplemental_tables_figures.zip › On-line Supplementary Material - Figure 2.pdf]

A high-protein total diet replacement increases energy expenditure and leads to negative fat balance in healthy, normal-weight adults. Camila L. P. Oliveira. Online Supplementary Material.

Supplementary Figure 2. Correlation between fat-free mass (FFM) and total energy expenditure (total EE, panels A and B), sleep energy expenditure (sleep EE, panels C and D), and resting energy expenditure on day 2 (REE, panels E and F) in males (n=24). Black squares (■) represent the high-protein total diet replacement (HP-TDR) condition and empty circles (○) represent the control (CON) condition.

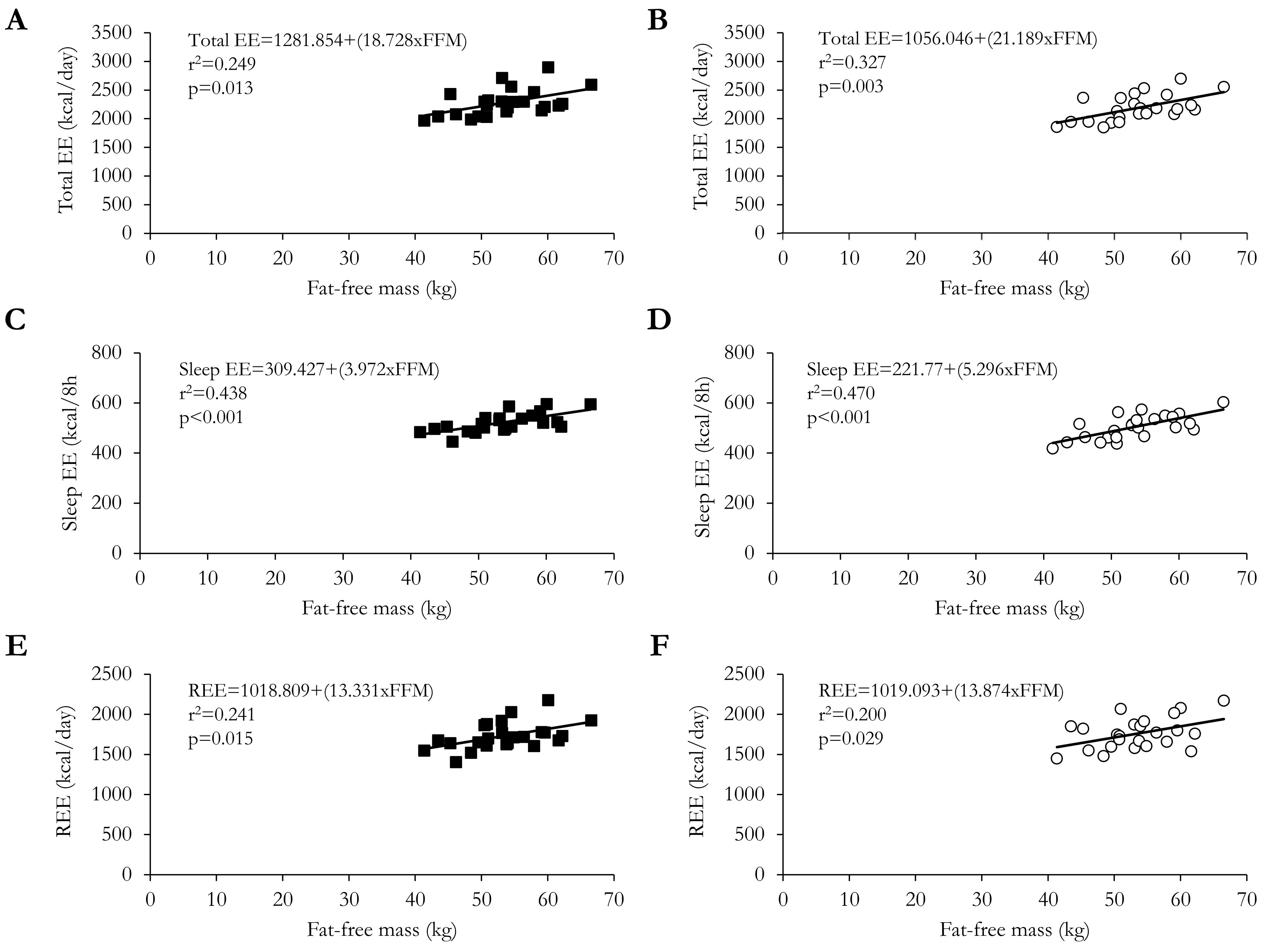

A
Total EE=1281.854+(18.728xFFM)
r2=0.249
p=0.013
Total EE (kcal/day)
Fat-free mass (kg)
B
Total EE=1056.046+(21.189xFFM)
r2=0.327
p=0.003
Total EE (kcal/day)
Fat-free mass (kg)
C
Sleep EE=309.427+(3.972xFFM)
r2=0.438
p<0.001
Sleep EE (kcal/8h
Fat-free mass (kg)
D
Sleep EE=221.77+(5.296xFFM)
r2=0.470
p<0.001
Sleep EE (kcal/8h)
Fat-free mass (kg)
E
REE=1018.809+(13.331xFFM)
r2=0.241
p=0.015
REE (kcal/day)
Fat-free mass (kg)
F
REE=1019.093+(13.874xFFM)
r2=0.200
p=0.029
REE (kcal/day)
Fat-free mass (kg)
