# Supplementary material for: A high-protein total diet replacement increases energy expenditure and leads to negative fat balance in healthy, normal-weight adults
Source: Am J Clin Nutr. 2020 Nov 18;113(2):476–87. doi: 10.1093/ajcn/nqaa283 (PMC7851826; doi:10.1093/ajcn/nqaa283)
Supplement: nqaa283_Supplemental_Tables_Figures [file nqaa283_supplemental_tables_figures.zip › On-line Supplementary Material - Table 1.pdf]

A high-protein total diet replacement increases energy expenditure and leads to negative fat balance in healthy, normal-weight adults. Camila L. P. Oliveira. Online Supplementary Material.

Supplementary Table 1. Nutrient content and ingredient list of the nutritional supplement used in the high-protein total diet replacement intervention.

Nutrients	Amount per 50 grams of powder
Energy (kcal)	180
Fat (g)	1.0
Saturated Fat (g)	0.5
Trans Fat (g)	0.0
Polyunsaturated Fat (g)	0.1
Monounsaturated Fat (g)	0.4
Cholesterol (mg)	3
Sodium (mg)	340
Potassium (mg)	500
Carbohydrate (g)	15
Fibre (g)	0.5
Sugars (g)	15
Protein (g)	27
Vitamin A (IU)	794
Vitamin C (mg)	16
Vitamin E (IU)	6
Vitamin B1 (mg)	5
Vitamin B2 (mg)	6
Vitamin B6 (mg)	7
Calcium (mg)	215
Iron (mg)	4.9

Ingredients: Soy protein isolate, honey, skim milk, yogurt powder, potassium chloride, magnesium carbonate, calcium citrate, vitamin C, niacin, color additive, riboflavin, vitamin E, zinc oxide, ferrous fumarate, manganese sulfate, calcium pantothenate, vitamin B2, vitamin B6, vitamin B1, vitamin A, folic acid, potassium iodide, sodium selenite, biotin, vitamin D3, and vitamin B12.
