# Supplementary material for: A high-protein total diet replacement increases energy expenditure and leads to negative fat balance in healthy, normal-weight adults
Source: Am J Clin Nutr. 2020 Nov 18;113(2):476–87. doi: 10.1093/ajcn/nqaa283 (PMC7851826; doi:10.1093/ajcn/nqaa283)
Supplement: nqaa283_Supplemental_Tables_Figures [file nqaa283_supplemental_tables_figures.zip › On-line Supplementary Material - Table 2.pdf]

A high-protein total diet replacement increases energy expenditure and leads to negative fat balance in healthy, normal-weight adults. Camila L. P. Oliveira. Online Supplementary Material.

Supplementary Table 2. Secondary analysis of metabolic blood markers during the HP-TDR and CON diets.

	HP-TDR			CON			Diet *x* Sex *x* Time[1]	Diet *x* Sex[1]	Diet *x* Time[1]
	**Fasting Day 1**	**Postprandial**	**Fasting Day 2**	**Fasting Day 1**	**Postprandial**	**Fasting Day 2**			
Glucose (mmol/L)[3]	4.8 ± 0.3	5.1 ± 0.4	4.7 ± 0.2	4.8 ± 0.3	4.9 ± 0.5	4.9 ± 0.3	0.512	0.523	0.321
Insulin (pmol/L)[3]	43.1 ± 15.4	62.8 ± 35.1	35.6 ± 13.8	44.8 ± 18.4	81.1 ± 50.2	37.1 ± 15.3	0.474	0.194	0.018
HOMA %B[3]	89.0 ± 20.5	-	78.7 ± 17.4	88.4 ± 21.7	-	76.2 ± 22.9	0.362	0.236	0.578
HOMA IR[3]	0.8 ± 0.3	-	0.6 ± 0.3	0.8 ± 0.3	-	0.7 ± 0.3	0.087	0.377	0.716
Glycerol (µM)[4]	27.5 ± 19.6	32.3 ± 23.0	19.3 ± 11.4	23.7 ± 14.4	47.6 ± 29.5	19.6 ± 12.0	0.781	0.648	<0.001
NEFA (µM)[4]	201.2 ± 191.6	115.2 ± 123.4	154.8 ± 139.5	182.1 ± 150.6	104.1 ± 88.4	145.7 ± 132.0	0.164	0.758	0.870
Lipid Panel[3]									
Total Cholesterol (mmol/L)	4.34 ± 0.73	4.33 ± 0.73	4.43 ± 0.78	4.3 ± 0.69	4.19 ± 0.72	4.28 ± 0.76	0.510	0.256	0.047
LDL Cholesterol (mmol/L)	2.41 ± 0.52	2.28 ± 0.49	2.54 ± 0.52	2.38 ± 0.49	2.13 ± 0.5	2.39 ± 0.5	0.759	0.536	0.005
HDL Cholesterol (mmol/L)	1.45 ± 0.43	1.45 ± 0.46	1.44 ± 0.48	1.43 ± 0.43	1.4 ± 0.44	1.4 ± 0.43	0.193	0.358	0.095
Non-HDL Cholesterol (mmol/L)	2.89 ± 0.58	2.88 ± 0.52	2.99 ± 0.58	2.88 ± 0.5	2.79 ± 0.52	2.88 ± 0.56	0.808	0.371	0.046
Triglyceride (mmol/L)	1.06 ± 0.42	1.31 ± 0.63	0.98 ± 0.34	1.08 ± 0.41	1.45 ± 0.57	1.08 ± 0.42	0.142	0.394	0.263

Data are presented as mean ± standard deviation.
[1] P-values represent diet *x* sex *x* time interaction and were detected with the use of a mixed analysis of variance.
[3] N=41 (N=17 females, N=24 males).
[4] N=42 (N=18 females, N=24 males).
Abbreviations: CON: control; HDL: high-density lipoprotein; HOMA %B: homeostatic model assessment of β-cell function; HOMA IR: homeostatic model assessment of insulin resistance; HP-TDR: high-protein total diet replacement; LDL: low-density lipoprotein.
